# Supplementary material for: Properties of Dietary Flavone Glycosides, Aglycones, and Metabolites on the Catalysis of Human Endoplasmic Reticulum Uridine Diphosphate Glucuronosyltransferase 2B7 (UGT2B7)
Source: Nutrients. 2023 Nov 28;15(23):4941. doi: 10.3390/nu15234941 (PMC10708323; doi:10.3390/nu15234941)
Supplement: Supplementary file 1 [file nutrients-15-04941-s001.zip › nutrients-2658609-supplementary.pdf]

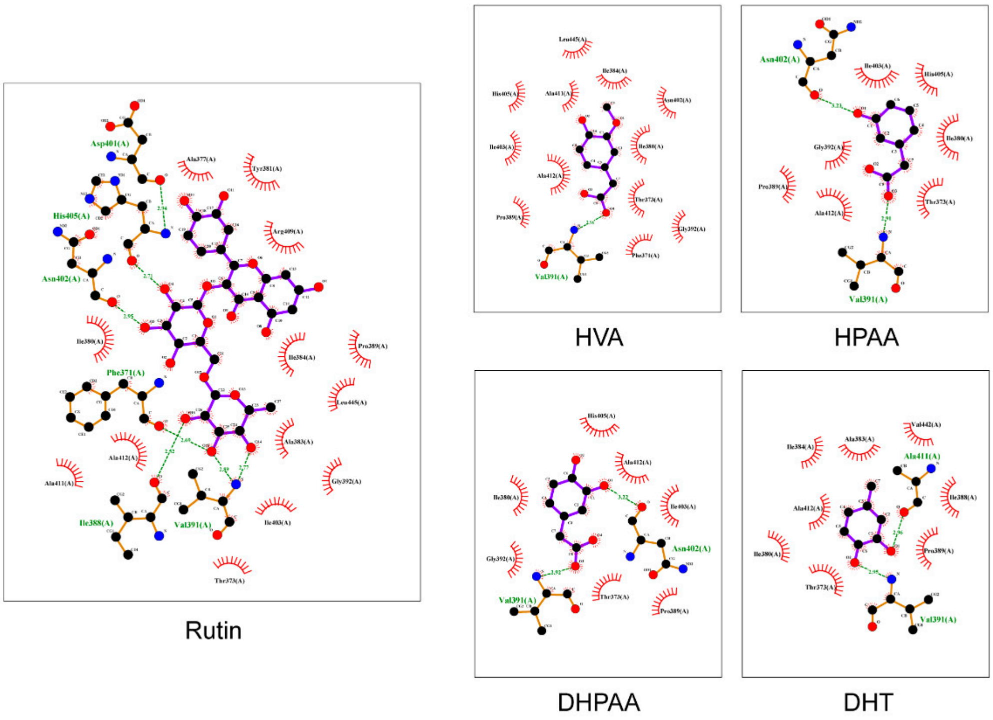

Figure S1. The Ligplot analysis of hydrophobic contacts between UGT2B7 and rutin/phenolic acid metabolites. The involved amino acids were Phe371, Thr373, Ala377, Ile380, Tyr381, Ala383, Ile384, Ile388, Pro389, Val391, Gly392, Asn402, Ile403, His405, Arg409, Ala411, Ala412, Val442, Leu445, respectively. Rutin and its phenolic acid metabolites were colored in purple, hydrogen bonds were represented by green dotted line, the numbers near the dotted line were represented length, non-ligand residues and corresponding atoms involved in hydrophobic contacts were represented in red comb shaped curves.

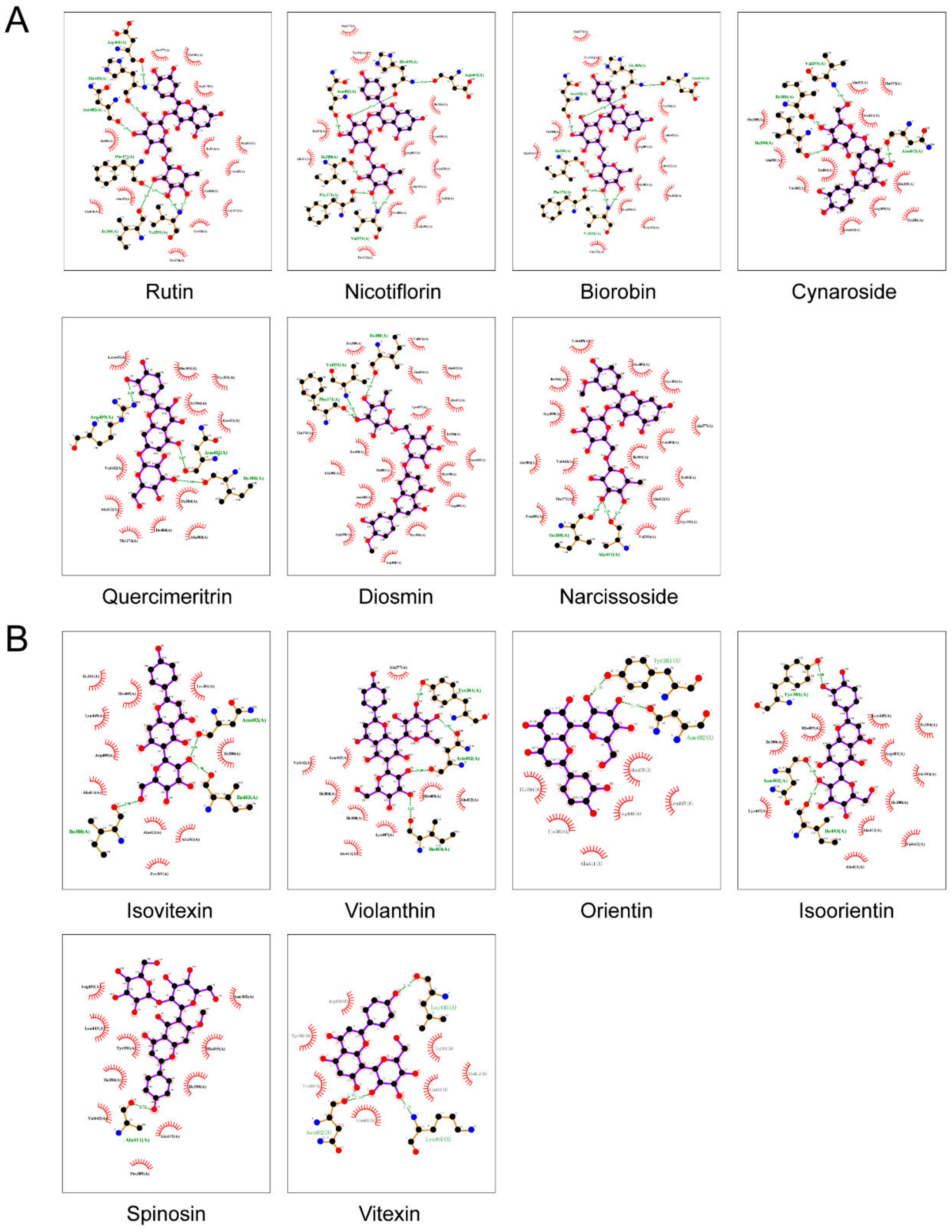

Figure S2. The Ligplot analysis of hydrophobic contacts between UGT2B7 and typical O-glycosylflavones (A) /C-glycosylflavones (B). The involved amino acids in (A) were Phe371, Thr373, Ala377, Ile380, Tyr381, Ala383, Ile384, Ile388, Pro389, Val391, Gly392, Asp398, Asp401, Asn402, Ile403, His405, Lys407, Arg409, Ala411, Ala412, Val442, Leu445, respectively. The involved amino acids in (B) were Ala377, Ile380, Try381, Ala383, Ile384, Ile388, Pro389, Asn402, Ile403, His405, Lys407, Arg409, Ala411, Ala412, Val442, Leu445, respectively. Typical O-glycosylflavones/C-glycosylflavones were colored in purple, hydrogen bonds were represented by green dotted line, the numbers near the dotted line were represented length, non-ligand residues and corresponding atoms involved in hydrophobic contacts were represented in red comb shaped curves.

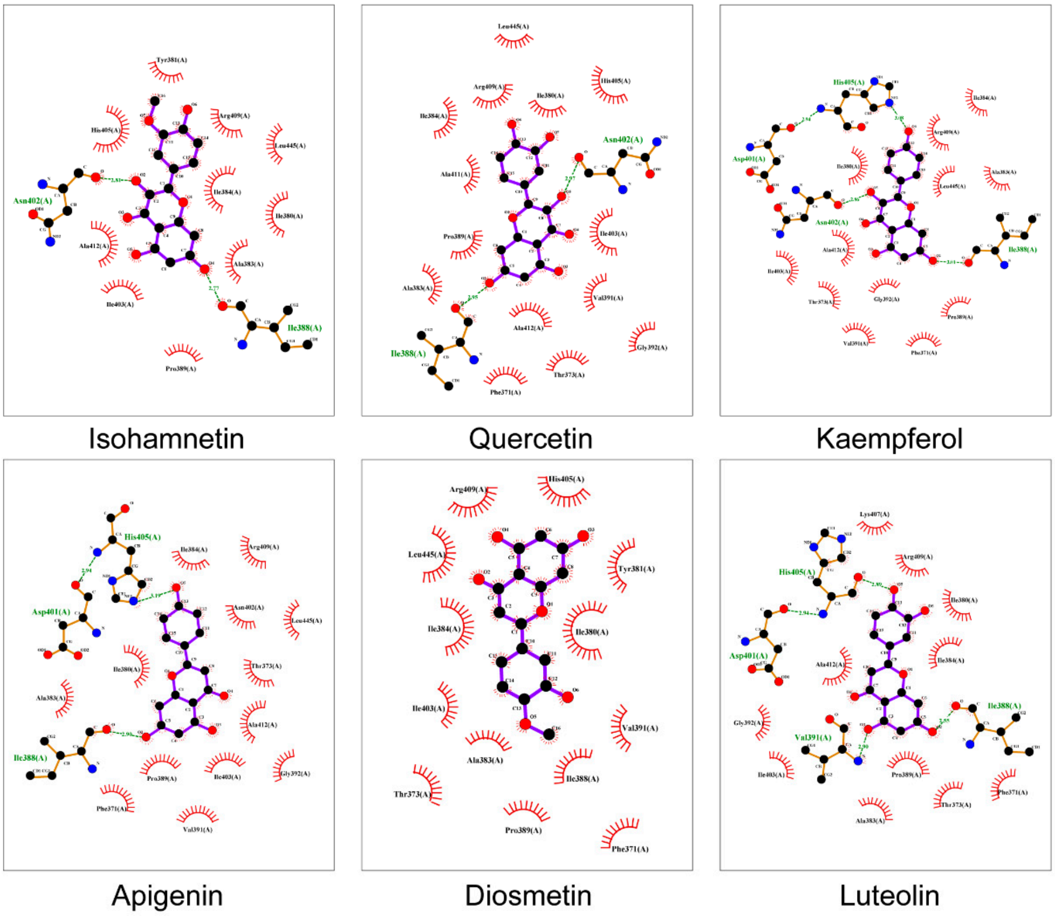

Figure S3. The Ligplot analysis of hydrophobic contacts between UGT2B7 and typical flavone glycosides. The involved amino acids were Phe371, Thr373, Ile380, Tyr381, Ala383, Ile384, Ile388, Pro389, Val391, Gly392, Asn402, Ile403, His405, Lys407, Arg409, Ala411, Ala412, Leu445, respectively. Typical flavonols were colored in purple, hydrogen bonds were represented by green dotted line, the numbers near the dotted line were represented length, non-ligand residues and corresponding atoms involved in hydrophobic contacts were represented in red comb shaped curves.

Table S1. Specific residues forming hydrophobic interactions with glycosides and aglycones.

Classification	Compound name	Residues forming hydrophobic interactions with glycosides	Residues forming hydrophobic interactions with aglycones
Flavonoid O-glycosides	Rutin	Phe371, Thr373, Ile380, Tyr381, Ala383, Ile384, Ile388, Pro389, Val391, Gly392, Ile403, Ala411, Ala412, Leu445	Ala377, Asn402, His405, Arg409, Leu445
	Quercimeritrin	Thr373, Ile380, Ala383, Ile388, Ile403, Ala411, Ala412, Val442, Leu445	Tyr381, Ile384, Asn402, His405, Arg409, Leu445
	Nicotiflorin	Phe371, Thr373, Ala377, Ile380, Tyr381, Ala383, Ile384, Ile388, Pro389, Val391, Gly392, Asn402, Ile403, His405, Arg409, Ala411, Ala412	Ala377, Ile380, Tyr381, Ile384, Asn402, His405, Arg409, Leu445

	Biorobin	Phe371, Thr373, Ala377, Ile380, Tyr381, Ala383, Ile384, Ile388, Pro389, Val391, Gly392, Asn402, Ile403, His405, Arg409, Ala411, Ala412	Ala377, Tyr381, Ile384, Asn402, His405, Arg409, Leu445
	Diosmin	Phe371, Tyr373, Ile380, Tyr381, Ala383, Ile384, Ile388, Pro389, Val391, Gly392, Asn402, Ile403, His405, Lys407, Arg409, ALA411, Ala412, Val442, Leu445	Tyr381, Asp398, Asp401, Asn402, His405, Arg409
	Narcissoside	Tyr373, Ile380, Ala383, Ile384, Ile388, Pro389, Val391, Gly392, Ile403, His405, Arg409, ALA411, Ala412, Val442, Leu445	Ala377, Tyr381, Asn402, His405, Arg409, Leu445
	Cynaroside	Tyr373, Ile380, Ala383, Ile384, Ile388, Pro389, Val391, ALA411, Ala412, Val442	Ile380, Tyr381, Ile384, Asn402, His405, Arg409, Leu445
Flavonoid C-glycosides	Vitexin	Ile380, Asn402, His405, Lys407, Arg409, ALA411, Ala412	Ile380, Tyr381, Ile384, Asn402, His405, Arg409, Leu445
	Isovitexin	Ile380, Ala383, Ile388, Pro389, Asn402, Ile403, His405, ALA411, Ala412	Ile380, Tyr381, Ile384, Asn402, His405, Arg409, Leu445
	Spinosin	Tyr381, Ile384, His405	Ile380, Ile384, Pro389, Asn402, His405, Arg409, Ala411, Ala412, Val442, Leu445
	Orientin	Ile380, Tyr381, Asn402, His405	Ile384, His405, Arg409, Ala411, Leu445
	Isoorientin	Ile380, Ala383, Ile384, Ile388, Asn402, Ile403, Lys407, ALA411, Ala412, Val442	Ile380, Tyr381, Ile384, Asn402, His405, Arg409, Leu445
	Violanthin	Ala377, Ile380, Tyr381, Ile384, Asn402, Ile403, His405, Lys407, ALA411, Ala412, Val442	Ile380, Tyr381, Ile384, Asn402, His405, Leu445

Table S2. IC50 value of the inhibition effect of compounds on UGT2B7 activity.

Components	IC50 Value
Rutin	74 μM
Nicotifiorin	98 μM
Isovitexin	Over 200 μM
Vitexin	Over 200 μM
HPAA	Over 200 μM
DHPAA	Over 200 μM
Aucubin	60 μM
Asperuloside	100 μM
